# Pleiotropic Roles of ABC Transporters in Breast Cancer

**DOI:** 10.3390/ijms22063199

**Published:** 2021-03-21

**Authors:** Ji He, Erika Fortunati, Dong-Xu Liu, Yan Li

**Affiliations:** 1 School of Science, Auckland University of Technology, Auckland 1010, New Zealand; ji.he@aut.ac.nz (J.H.); pjz3731@autuni.ac.nz (E.F.); 2 The Centre for Biomedical and Chemical Sciences, School of Science, Faculty of Health and Environmental Sciences, Auckland University of Technology, Auckland 1010, New Zealand; dongxu.liu@aut.ac.nz; 3 School of Public Health and Interprofessional Studies, Auckland University of Technology, Auckland 0627, New Zealand

**Keywords:** ABC transporter, breast cancer (BC), multidrug resistance (MDR), metastasis

## Abstract

Chemotherapeutics are the mainstay treatment for metastatic breast cancers. However, the chemotherapeutic failure caused by multidrug resistance (MDR) remains a pivotal obstacle to effective chemotherapies of breast cancer. Although in vitro evidence suggests that the overexpression of ATP-Binding Cassette (ABC) transporters confers resistance to cytotoxic and molecularly targeted chemotherapies by reducing the intracellular accumulation of active moieties, the clinical trials that target ABCB1 to reverse drug resistance have been disappointing. Nevertheless, studies indicate that ABC transporters may contribute to breast cancer development and metastasis independent of their efflux function. A broader and more clarified understanding of the functions and roles of ABC transporters in breast cancer biology will potentially contribute to stratifying patients for precision regimens and promote the development of novel therapies. Herein, we summarise the current knowledge relating to the mechanisms, functions and regulations of ABC transporters, with a focus on the roles of ABC transporters in breast cancer chemoresistance, progression and metastasis.

## 1. Chemoresistance in Breast Cancer

Worldwide, breast cancer (BC) is the most commonly diagnosed cancer and the leading cause of cancer death, with an estimated 2.3 million new cases and 685,000 deaths in 2020 [1]. The systemic treatment of breast cancer includes cytotoxic-, hormonal- and immune-therapeutic agents, which are utilised in adjuvant, neoadjuvant and metastatic settings [2]. Anthracycline- and taxane-based regimens as adjuvant chemotherapies have been shown to significantly improve the disease-free survival (DFS) and overall survival (OS) rates in high-risk, early-stage breast cancer patients [3,4]. These regimens have been preferred for hormone receptor-negative patients with metastatic breast cancer (MBC) [5]. However, due to the phenomenon of drug resistance causing reduced tumour chemosensitivity over time, patients receiving anthracycline or taxane-based regimens may not have a sustained response to these agents, despite displaying initial positive responses. The development of drug resistance is a frequent problem in the management of breast cancer; many patients’ tumours are resistant to the therapeutic agents by the time of disease recurrence, leading to the average five-year survival rate of patients with stage 0 or stage I, stage II and stage III MBC at 100%, 93%, 72% and 22%, respectively [6]. These data underscore drug resistance as a dominant influence on the survival of breast cancer patients after breast mastectomy, metastasis or recurrence. Once metastasis or drug resistance develops, the treatment options are very limited, and the possibility of a cure is practically nonexistent.

The chemoresistance displayed by cancer cells is primarily attributed to multidrug resistance (MDR). The resistance of tumours to one drug or drug combination can trigger cross-resistance to other structurally or mechanistically unrelated chemotherapeutic compounds [7,8]. This may explain why targeted multidrug combination regimens do not exhibit superior efficacy to single-agent therapies. In the current clinical settings, MDR remains a significant impediment to successful chemotherapy, leading to the relapse and progression of most malignant tumours. The mechanisms of action mediating the development of MDR appear to be quite complicated, including but not limited to increased extracellular drug efflux, decreased intracellular drug uptake, alterations in drug metabolism, mutations of drug targets and inactivation of death signalling pathways [9]. These mechanisms can generally be divided into two broad groups: intrinsic or acquired [9]; intrinsic resistance is when MDR is naturally expressed in tumours, while acquired resistance is when MDR develops as a result of chemotherapy. Thus, the effectiveness of chemotherapeutic agents might be limited by intrinsic resistance before treatment begins and can worsen during the treatment due to acquired resistance.

**Figure 1:**
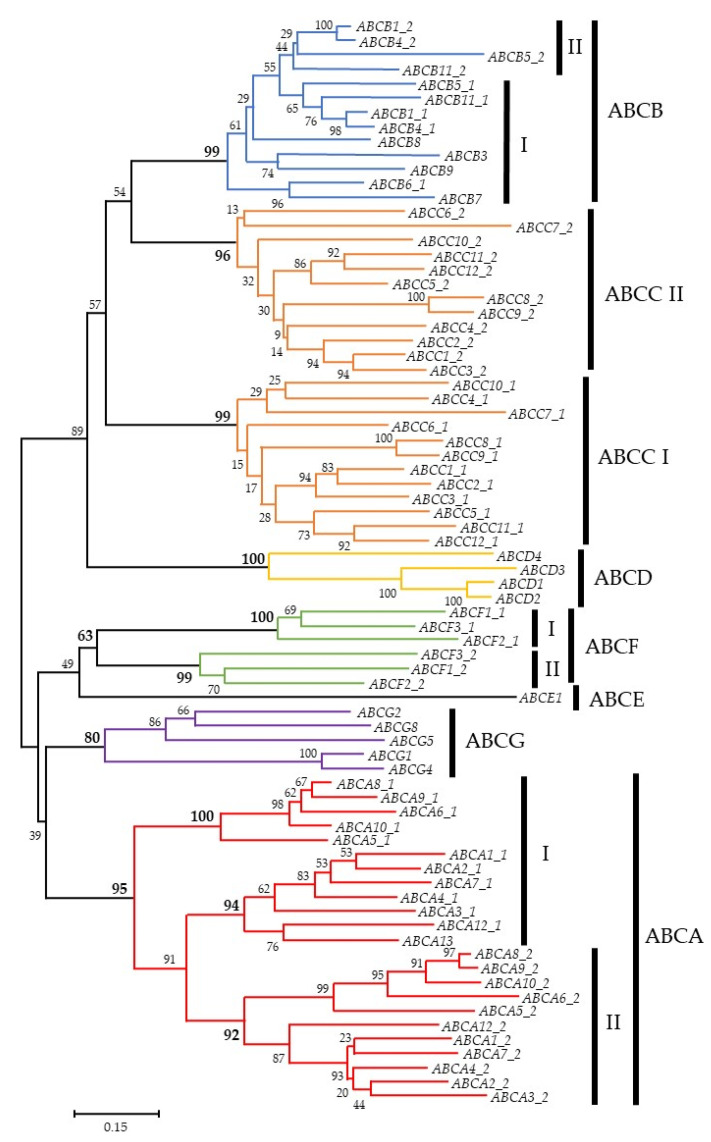
Phylogenetic tree of the human ATP-Binding Cassette (ABC) genes. The alignments are based on data from reference [19]. Some of the analysed proteins contain two ATP-binding domains (I and II), whereas others contain only one ATP-binding domain. The number at the branch of the nodes represents the value from 100 replications.

**Figure 2:**
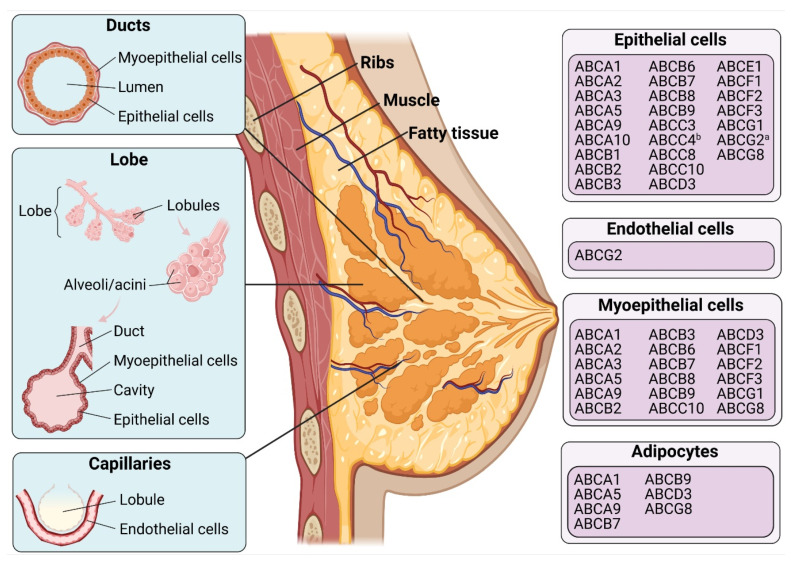
Distribution of ABC proteins in human mammary glands. “a” indicates the apical membrane, and “b” indicates the basolateral membrane. Data were extracted from The Human Protein Atlas database (http://www.proteinatlas.org; Last access date: 1 March 2021) and references [20,21,22,23]. Figures were created with BioRender.com (accessed on 22 March 2021).

After the ground-breaking discovery of MDR in 1976 [10], membrane proteins have been extensively researched as potential causes of drug resistance. In humans, 48 genes encoding the ATP-Binding Cassette (ABC) transporter superfamily have been identified and phylogenetically divided into seven subfamilies (i.e., ABC subfamily A to G). Based on their sequence homology and domain organisation (Figure 1) [11,12], ABC transporters, such as the extensively studied multidrug resistance protein 1 (MDR1 a.k.a. P-glycoprotein, P-gp, and ABCB1), MDR-associated protein 1 (MRP1 a.k.a. ABCC1) and breast cancer resistance protein (BCRP a.k.a. ABCG2), enable cancer cells to elude “hazardous substances” by extruding them extracellularly through a process known as drug efflux [13]. ABC transporters contain a complicated translocation system responsible for several physiological functions, including the unidirectional movement of diverse compounds across the phospholipid bilayer of cellular membranes [14]; regulating intracellular levels of hormones, lipids, ions, xenobiotics and other small molecules [15,16] and maintaining physiological homeostasis via the intracellular regulation of organelles, such as the mitochondrion, lysosome, endoplasmic reticulum and Golgi apparatus [16]. A significant pharmacological implication of these protective roles is the ABC transporter-mediated efflux of various drugs, including anticancer agents against the concentration gradient, leading to decreased cellular accumulation and, thus, MDR. In fact, only 20 or so ABC transporters are presumably associated with a drug-resistance phenotype and lead to a transient or even incomplete response to anticancer pharmaceuticals [14]. These ABC transporters exhibit partially overlapping substrate specificity, because they share genetic sequence and structural homology [17,18]. Despite the extensive research into the expression and localisation profiles of ABC transporters in human breast tissues (Figure 2), their clinical implications have been controversial and unclear. This review summarises the structure, clinical insights and pharmacokinetics and drug-resistant functions of the most representative ABC transporter, ABCB1, in breast cancer and further discusses the role of ABC transporters in breast cancer progression and metastasis.

## 2. The Role of ABCB1 in Breast Cancer Chemoresistance

### 2.1. Structure and Mechanism of ABCB1

The first ABC transporter to be identified and published was MDR1 (multidrug resistance protein 1 a.k.a. P-glycoprotein, P-gp, and ABCB1), which is distributed in nearly all normal tissues at various levels but is found overexpressed in many tumours [9]. P-gp was originally isolated by Juliano and Ling [10] from the plasma membranes of Chinese hamster ovary cells; it was referred to as “P-glycoprotein” due to its ability to reduce the drug permeability in resistant cells. The gene encoding this transporter in an animal model and the human homologue was subsequently identified in respective studies [24,25]. From then on, the human gene encoding P-gp was termed and classified as ABC subfamily B member 1 (*ABCB1*) [24,25]. ABCB1 is predominantly expressed in numerous apical membranes of human epithelial cells, including the gastrointestinal tract, pancreatic ductulus, liver and kidney, as well as in the endothelial cells of the blood–brain barrier (BBB) [26]. This widespread distribution highlights the role of ABCB1 in protecting the body from xenobiotics and other natural toxins. A consequence of these protective roles is that the presence of active ABCB1 can typically interfere with the net absorption and penetration of many therapeutic compounds into normal healthy cells within the intestine, kidneys, liver and BBB [27]. For instance, evidence shows that ABCB1 could restrict the oral bioavailability of the substrate drug paclitaxel by impeding intestinal absorption [28]. Similarly, the oral bioavailability of larotrectinib, a neurotrophic tropomyosin receptor kinase (NTRK) inhibitor, was markedly limited in wild-type mice compared to *Abcb1a/b^−/−^* mice by limiting the net absorption or regulating the hepatobiliary elimination or a combination of both processes [29]. The brain and testis penetration of larotrectinib was also restricted by Abcb1a/b [29]. ABCB1 also limits the brain penetration of many other drugs, such as tivozanib (a vascular endothelial growth factor receptor inhibitor); galunisertib (a transforming growth factor-beta receptor inhibitor); fisogatinib (a fibroblast growth factor receptor 4 inhibitor); osimertinib (an epidermal growth factor receptor inhibitor) [30,31,32] and CDK4/6 inhibitors (palbociclib, ribociclib and abemaciclib), in murine models [33]. Additionally, accumulating evidence implies that ABCB1 and, probably, other ABC transporters can influence the pharmacokinetics and the contribution to the therapeutic efficacy of substrate drugs between individual patients. Clinical testing for these genetic polymorphisms in human ABCB1 transporter genes could provide a practical tool for predicting the drug response and individualisation of therapy.

**Figure 3:**
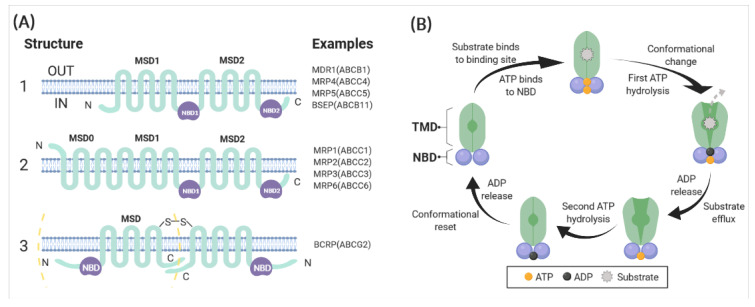
(**A**) Basic structure of ABC transporters. The three different structures of ABC transporters known to render drug resistance. (1) ABC transporters such as ABCB1 and ABCC4 possess 12 transmembrane regions and two ATP-binding sites. (2) ABCC1, 2, 3 and 6 are similar in structures in that they have two ATP-binding regions. Compared with ABCB1, they have an amino-terminal end extension that contains five transmembrane regions, with a total of 17 transmembrane regions. (3) ABCG2 “half-transporter” and ABCG2 homodimer. The “half-transporter” ABCG2 just contains six transmembrane regions and one ATP-binding site. This kind of transporter is thought to function as either a homodimer or an oligomer (Chen, Manautou [38]). (**B**) Schematic representation of the proposed pumping action of ABCB1. The substrate of ABCB1 binds to the binding pocket in the transmembrane domains (TMDs), and ATP binds to the two ATP-binding sites in the nucleotide-binding domains (NBDs). Then, the first ATP hydrolysis provides energy for the generation of a conformational change from which the substrate is released. This is followed by the hydrolysis of the second ATP, which resets the altered conformation, allowing repeating of the efflux process (Robey, Pluchino [16]). Figures were created with BioRender.com (accessed on 22 March 2021).

Current evidence supports a topology model in which ABCB1 has two intracellular ATP-binding sites and 12 transmembrane segments with a highly N-glycosylated first extracellular loop (Figure 3A) [26]. These regions and sites form four domains, including two cytoplasmic nucleotide-binding domains (NBDs) and two transmembrane domains (TMDs). The current knowledge demonstrates that NBDs are highly conserved domains that are structurally and functionally similar throughout ABC transporter families. NBDs are connected to each other in a head-to-tail model to form a “sandwich dimer” that consists of two composite nucleotide-binding sites, allowing ATP to bind and hydrolyse at the ATP-binding sites [34]. TMDs, however, are highly heterogeneous, enabling ABC transporters to recognise and translocate a wide spectrum of substrates across membranes with the energy of ATP hydrolysis, irrespective of the prevailing concentration gradient [19]. In order to bind and transport plenty of engaged substrates, basal ATP hydrolysis drives the continuous conformational changes of ABC transporters [35]. To summarise, intracellular molecules bind to TMDs due to their high-affinity conformations. Following this, ATP binds to the NBDs of the ABC transporter structure. These binding events stimulate the ATPase activity of ABCB1, causing ATP hydrolysis and the generation of conformational changes from which the molecule is released. The altered conformation can be restored by the energy of ATP hydrolysis at the second ATP-binding site. Lastly, the transporter resumes its high-affinity conformation, allowing the repetition of the excretory process (Figure 3B) [13,35,36,37].

### 2.2. Expression and Function of ABCB1 in Breast Cancer

The expression of ABCB1 in breast tumours significantly varies between individuals. Studies have shown that ABCB1 is highly expressed in the lymph node metastases of invasive ductal breast cancer patients [39]. For instance, Trock and Leonessa [40] conducted a meta-analysis of 31 studies from 1989–1996 to examine the ABCB1 expression in breast tumours and found that 41% of the breast tumour samples were positive for ABCB1 expression. A series of imaging studies in vivo using ^99m^Tc-sestamibi (technetium-99m sestamibi a.k.a. Cardiolite), an ABCB1 substrate, further confirmed that ABCB1-mediated drug extrusion is enhanced in some patients with breast carcinomas [41,42,43]. The incidence of ABCB1 expression was higher in patients receiving cytotoxic chemotherapy, and its expression in breast tumours was associated with treatment failure and poor chemotherapeutic response [40]. In a study involving tissue samples from 712 Brazilian women who received mammary surgery, a lower expression of ABCB1 estimated using immunohistochemistry was linked to triple-negative breast cancer (TNBC) and a worse prognosis [20]. Of the 606 nonmalignant mammary tissue samples adjacent to the tumour area that were examined, 98.8% of cases showed a positive reaction for ABCB1 in mammary ducts and acini, and only 0.8% showed a negative reaction in both the cancer cells and adjacent nonmalignant breast tissue, indicating the high prevalence of ABCB1 expression in mammary tissues. The ABCB1 negativity was also associated with hypertensive status, large tumour size and low lymph node status [20]. While *ABCB1* expression was significantly downregulated in post-treatment breast tumour specimens compared with non-neoplastic tissues [44], ABCB1 was related to resistance to 5-fluorouracil; Adriamycin and cyclophosphamide (FAC)/5-fluorouracil, epirubicin and cyclophosphamide (FEC) chemotherapy in another study involving 59 patients [45]. The expression of *ABCB1* is significantly correlated to epigenetic factors. For example, the hypermethylation of CpG island regions covering the distal promoter of the *ABCB1* gene might be linked with a lower *ABCB1* transcript expression level and longer median OS in breast and ovarian cancer patients [46]. In addition, naturally occurring genetic variants (a.k.a. single-nucleotide polymorphisms; SNPs) of *ABCB1* may influence the function and expression of this transporter, thus causing variations in the intestinal absorption, the elimination and the penetration of drugs into the cells. The interindividual variability in the pharmacokinetics and the pharmacodynamics of many drugs related to ABCB1 is a result of this phenomenon [47]. The SNPs rs1045642, rs1128503 and rs2032582 in *ABCB1* were found to contribute to the altered pharmacokinetics of doxorubicin in Asian breast cancer patients [48]. The GT genotype of the rs2032582 variant in *ABCB1* was further linked to improved breast cancer-specific survival (BCSS) compared with the GC/GA genotype in a study of 879 breast cancer patients who received FEC chemotherapy [49]. Moreover, the *ABCB1* polymorphism C1236T was significantly associated with docetaxel response in a study of 129 locally advanced South Indian breast cancer patients [50]. Apart from the association of *ABCB1* SNPs with drug efficacy, the variants also play a role in chemotherapeutic side effects. For example, Hertz and Caram [51] reported that *ABCB1* SNP rs1045642 3435C>T displayed a cardioprotective effect in a study of 166 breast cancer patients treated with doxorubicin. These studies suggest that the role of ABCB1 in mediating chemoresistance in breast cancer has not been clearly uncovered. It is potentially implicated in breast cancer MDR, but the extent to which ABCB1 alters chemoresistance is controversial and likely dependent on its expression. *ABCB1* expression might be associated with breast cancer molecular characteristics, the hypertensive status, tumour size, lymph node status, ethnicity, posttranscriptional events, epigenetic factors and *ABCB1* gene variants. Besides, the detection of ABCB1 in breast carcinoma also needs to be compared with its expression in normal mammary tissues adjacent to tumour area, especially considering the high prevalence of ABCB1 expression in breast tissue. Intriguingly, gestational age was found to affect ABC transporter expression (e.g., ABCB1 and ABCG2) in human placenta [52], while ABCG2 was upregulated during lactation [53]. Hence, the expression pattern of ABCB1 and many other ABC transporters in breast tissue during gestation, lactation and even menopause may merit attention when evaluating the expression of these pumps in breast cancer. A comprehensive analysis of ABCB1 expression in more specific breast cancer subsets is required to expand the current knowledge about its role in breast cancers.

The overexpression of ABCB1 has been correlated with chemoresistance in many cancer cell lines, such as kidney, colon, adrenal, pancreas, liver and breast cancers [40,54]. ABCB1 is capable of transporting hydrophobic substrates that are either neutral or positively charged [27]. The hydrophobic property of ABCB1 substrates probably enables them to passively and effectively diffuse across membranes [27]. The substrates (Table 1) are normally organic molecules ranging in size from less than 200 Da to almost 1900 Da, containing diverse cytotoxic chemotherapeutic compounds, such as anthracyclines and taxanes [27]. To complicate matters, these regimens have been widely used in treating breast cancer [55]. ABCB1 protein was significantly overexpressed in paclitaxel-resistant SKBR3 and MCF7 breast cancer cells. Yet, the silencing of the *ABCB1* gene partially resensitised those cells [56]. ABCB1 was also found to render eribulin [57] and cisplatin resistance [58] in MCF7 and MDA-MB-231 breast cancer cells. Similarly, Mechetner and Kyshtoobayeva [59] found that the expression of ABCB1 was strongly correlated with doxorubicin and Taxol resistance in vitro by using patient-derived breast cancer cells. In vivo mouse breast tumour studies suggest that even a moderate increase in murine *Abcb1a* and *Abcb1b* gene expression has been implicated in doxorubicin, docetaxel, topotecan and olaparib resistance, which could be reversed by the P-gp inhibitor tariquidar [60,61,62,63]. Recent in vitro studies have demonstrated that some molecularly targeted drugs (e.g., imatinib, erlotinib, sunitinib and nilotinib) can be transported by ABCB1 and ABCG2 [64]. Olaparib is a newly approved poly (ADP-ribose) polymerase (PARP) inhibitor, indicated for the treatment of cancers that exhibit DNA repair defects, including *BRCA1/2*-mutated breast cancers. Olaparib was a substrate of ABCB1 in a cancer cell model [65]. In an in vivo study, the murine *Abcb1a* and *Abcb1b* genes could mediate olaparib resistance in a mouse model. However, the tumours still developed resistance after long-term exposure to olaparib in *Abcb1a/b^−/−^* mice, indicating the involvement of other mechanisms in olaparib resistance [66,67]. This might be partially due to the aberrant expression of *SPAG5* (sperm-associated antigen 5) in breast tumour cells, as we previously reviewed [68]. SPAG5 could confer resistance to olaparib by either upregulating homologous recombination (HR) DNA repair proteins RAD51 and BRCA1/2 or shortening of the S phase duration in which olaparib-provoked DNA lesions occur [68]. Additionally, CDK7 as a key modulator in both the regulation of transcription and the cell cycle has been a potentially valuable cancer drug target. ABCB1 was identified as a common mechanism of resistance to the CDK7 inhibitors, ICEC0942 and THZ1, in MCF7 breast cancer cells [69]. Moreover, ABCB1 was reported to limit the intracellular retention of the photosensitising agent benzoporphyrin derivative (BPD) in photodynamic therapy (PDT) in the MCF7 breast cancer cell line [70]. ABCB1 could confer a resistance to PDT by exporting the light-absorbing photosensitisers. The nontoxic photosensitisers as light-sensitive compounds could be activated by light excitation and subsequently induce the generation of superoxide anion radicals and reactive singlet oxygen molecules (e.g., O_2_, H_2_O_2_ and ·OH) that confer toxicity to nearby targets [71].

### 2.3. Inhibition of ABCB1 in Breast Cancer

Given the profound impact of ABCB1 on the pharmacokinetic profiles of various anticancer drugs, it is of great interest to search for efficacious inhibitors. Many agents have been reported as modulators in counteracting ABCB1-induced MDR, such as first- (e.g., verapamil, cyclosporine A and quinine); second- (e.g., valspodar, dofequidar and biricodar) and third-generation (e.g., elacridar, laniquidar, zosuquidar and tariquidar) ABCB1 inhibitors [78]. Tariquidar, a third-generation ABCB1 inhibitor, has obtained promising results, which contributed to a greater ABCB1 inhibition than the first- and second-generation inhibitors at the human BBB. The brain uptake of ABCB1 substrates (11)C-N-desmethyl-loperamide and (R)-(11)C-verapamil showed approximately five- and three-fold increases in healthy human volunteers with a concurrent administration of tariquidar, respectively [79,80]. The murine mammary tumours also showed an increase in the uptake of (R)-(11)C-verapamil after the administration of tariquidar in mice [81]. Lapatinib, a tyrosine kinase inhibitor, increased the intestinal absorption of oral intake digoxin (a drug substrate of ABCB1) in patients with HER2-positive metastatic breast cancer [82]. However, the inhibition of solely ABCB1, in some cases, is not sufficient for increasing the tissue distribution of many chemotherapeutics. This is likely due to the broadly overlapping substrate specificity of the ABC transporters. Inhibitors targeting multiple ABC transporters are therefore considered to be more effective. Elacridar, a tyrosine kinase inhibitor, is an example of a dual ABCB1/ABCG2 inhibitor that increased the concentration of its substrate, erlotinib, at the BBB in a mouse model [83], but it was restricted by its low oral bioavailability in humans [84]. When it comes to the human body, Erlotinib was reported to inhibit ABCB1 and ABCG2 at the human BBB but at a supratherapeutic-dose [85]. Nevertheless, the clinical application of ABC transporter inhibitors in overcoming MDR is restricted by several aspects. The clinical application of ABC transporter inhibition in cancer patients might potentially induce serious side effects, such as cardiotoxicity. Indeed, ABCB1 can be expressed by healthy cells and play significant protective roles in barrier organs and tissues. Yet, suppressing these protective functions may result in harmful side effects, outweighing any potential effects against chemoresistance. The systemic inhibition of ABC transporter activity is also thought to interfere with vital immune functions and with the efficacy of anticancer immune responses. ABC transporters expressed in immune cells could influence the development and the functionality of T cells and dendritic cells through regulating the secretion of immune regulatory molecules that are associated with intracellular signalling, mediating cell differentiation and migration [86].

Unfortunately, in clinical studies, the administration of these ABCB1 inhibitors, along with anticancer drugs, showed poor results, with no benefits on the OS and DFS in patients bearing different types of cancers, including breast cancer [78]. This indicates that ABC transporters may not play a key role in clinical MDR. The use of cell or mouse models with high ABC transporter expression levels may lead to the overestimation of the role they play in chemoresistance. Especially in gene knockout studies, the CRISPR-cas9-induced off-target effects and the production of truncated protein isoforms that skip the edited exon always limit CRISPR technology and lead to unexpected knockout lines [87,88]. Off-target effects could lead to the unexpected editing of other genes and compromise the univariate analysis of the target gene [87,88]. However, commonly used short-range PCR assays are unable to detect and examine what is truly occurring in knockout cell lines [87,88]. The production of truncated protein isoforms could remain the cellular function of target proteins and potentially involve other unknown roles. Yet, extensively used immunoblotting assays are less sensitive and more biased in detecting protein isoforms due to the dependence on the epitope of the antibody [87,88]. Whole-genome sequencing and high-resolution mass spectrometry illustrate a solution to these disadvantages, but the expensive costs and complicate techniques appear to be the major obstacles [87,88]. In addition, ABC transporters are usually mediated by the same set of transcriptional activators with other proteins in a defined network. In the case of a member of this network becoming upregulated, the driving factor of ABC transporter upregulation might be the altered expression of this network rather than the exposure to chemotherapeutics [89]. The aberrant expression of such ABC transporters, in this case, is thought to be a byproduct of genetic alterations. In order to identify the involvement of the upregulated ABC transporters in acquired drug resistance, the rearrangement status of the coding gene is taken into account [89]. Christie and Pattnaik [90] detected *SLC25A40-ABCB1* transcriptional fusions in 9 out of 33 recurrent breast cancer patient samples in a recent study. Such transcriptional fusions (rearrangements) between *ABCB1* and its fusion partners (e.g., *SLC25A40* and *CNOT4*) provide *ABCB1* with a more active promoter at the 5′ end of its transcript and, thus, upregulate *ABCB1* expression in high-grade serous ovarian cancer (HGSC) and end-stage breast cancer patients [90]. A fusion event was found to either exist alone or cooccur with other fusions and was only detected in patients who received chemotherapies that are known ABCB1 substrates [90]. Fusion partners that are located on the same chromosome as *ABCB1* were more likely to recombine with *ABCB1*, but the recombination of *ABCB1* with fusion partners was still determined by the chromosomal proximity to *ABCB1*, transcriptional level and orientation and position relative to *ABCB1* [90]. Nevertheless, this correlation of *ABCB1* with drug resistance was still potentially ascribed to the co-amplification of an adjacent gene next to *ABCB1* (e.g., *ABCB4*), considering the broadly overlapping substrate specificity of ABC transporters [89]. To further understand the role of ABC transporters in clinical MDR, the ABC transporter expression and their underlying mechanisms need to be precisely identified. Any error in evaluating the ABC transporter expression level of patients and the mechanisms may lead to the misunderstanding of the true clinical benefits of the ABC transporter inhibition. For example, it has been suggested that ABCB1 expression is the highest in tumour-associated macrophages in breast stromal compartments, rather than the tumour samples themselves [16,91]. The heterogeneity of breast tumours also impacts the accuracy of measuring the ABC transporter expression levels in clinical settings. Clinically testing ABC transporters using single-cell transcriptome sequencing may allow the inference of cell type composition and a more accurate quantitation of gene transcripts in breast tumour tissues [92,93]. Additionally, drug resistance is usually derived from the combined activity of different ABC transporters and, presumably, many other mechanisms during cancer progression. As a result, other signalling pathways may alternatively compensate for the lowered drug resistance in tumour cells induced by ABC transporter inhibition. Simply evaluating the role that a single type of ABC transporter plays in breast cancer MDR might not mirror the entire underlying mechanisms behind MDR [94]. Therefore, the expression level of ABC transporters, along with other molecular characteristics of breast cancer patients, need to be carefully screened, selected and classified in future clinical studies.

The above evidence demonstrates that ABCB1 alone may not be an ideal biomarker to predict patients’ response to first-line treatments for primary breast tumours. However, some ABC transporters are found to play an opposing role in cancer chemoresistance. Rather than increasing the survival of cancer cells in the presence of therapeutic agents, ABC transporters could enhance the susceptibility of cancer cells through a phenomenon termed collateral sensitivity (CS). The term CS was first proposed in a study by Szybalski and Bryson in 1952 to describe the hypersensitivity of drug-resistant *Escherichia coli* to other structurally and/or mechanistically unrelated agents [95]. CS is a type of synthetic lethality in which the genetic alterations accrued that confer resistance to one agent sensitises the cells to a second agent. This phenomenon results in novel sensitivity to the identified chemotherapeutics irrespective of the presence of MDR mechanisms, overcoming problems with MDR cells [96]. For example, doxorubicin-resistant MCF7/Adr cells overexpressing ABCB1 showed up to 300-fold collateral sensitivity to a series of synthesised 10-*O*-dihydroartemisinin (DHA; derivative of artemisinin) derivatives compared with the parental line [97]. This selective targeting effect was likely derived from G1 phase arrest via the downregulation of the cell cycle regulatory proteins cyclin D1 and B1 [97]. Given that the MDR-induced relapse and progression of most malignant tumours remains a significant impediment to successful chemotherapy, the exploitation of MDR by CS compounds provides a novel strategy against MDR tumours. Therefore, the capability of CS compounds to target ABC transporter overexpressing cells is of intense interest, and more systemic studies are expected to uncover the molecular signalling pathways that are involved in breast cancer. 

## 3. The Role of ABC Transporters in Breast Cancer Development and Metastasis

### 3.1. ABC Transporters and Breast Cancer Development

**Figure 4:**
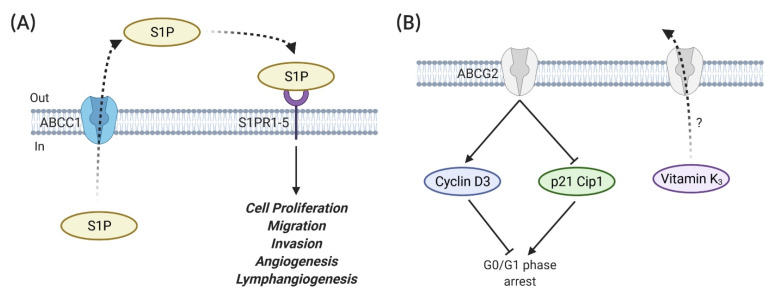
The role of ABC transporters in in vitro proliferation. (**A**) ABCC1 is involved in the export of sphingosine-1-phosphate (S1P). The binding of extracellular S1P to a family of G protein-coupled receptors (S1PR1-5) facilitates cell proliferation, migration, invasion, angiogenesis and lymphangiogenesis [99,100]. (**B**) ABCG2 inhibits G0/G1 arrest through the upregulation of cyclin D3 and downregulation of p21 Cip1. ABCG2 may also participate in the cell cycle via the translocation of endogenous substrates that relate to the cell cycle, such as vitamin K_3_ [98]. Figures were created with BioRender.com (accessed on 22 March 2021).

Apart from their drug efflux abilities, emerging evidence suggests the contributions of ABC transporters to cancer biology in either a substrate efflux dependent or independent manner. ABC transporters possibly have more roles in cancer development, leading to cancer cell proliferation, differentiation, invasion and migration. Chen and Liu [98] linked ABCG2 to cell cycle progression in mitoxantrone-resistant cells (MCF7/MX); the knockdown of *ABCG2* remarkably suppressed the proliferation of MCF7/MX cells via G0/G1 phase arrest (Figure 4B) [98]. This G0/G1 growth arrest was associated with the downregulation of cyclin D3 and upregulation of p21 Cip1. The authors also proposed that the ABCG2-enhanced cell proliferation in MCF7/MX cells could be attributed to the export of endogenous substrates (e.g., vitamin K_3_) that correlate to the cell cycle [98]. However, the ABCG2-enhanced cell proliferation was not observed in ABCG2 overexpressing cells with both plasmid transfection and retrovirus transduction, which was attributed to a phenomenon known as oncogene addiction [98]. Similarly, Low and Shabir [99] discovered that ABCC1 was correlated to breast cancer proliferation in siRNA knockdown cell models. Its expression was strongly correlated to high-grade breast carcinomas in breast cancer patients [44]. However, the mechanisms whereby ABCC1 exerts its role in cell proliferation are still controversial. A possible mechanism of ABCC1-regulated tumour growth is the elevated production and export of S1P (sphingosine-1-phosphate) by ABCC1 (Figure 4A). The bioactive sphingolipid mediator S1P can bind to a family of G protein-coupled receptors (S1PR1-5) to promote cell proliferation, migration, invasion, angiogenesis and lymphangiogenesis. ABCC1 can thus mediate these features of cancers by exporting intracellularly generated S1P out of cancer cells and contribute to this “inside-out” signalling [100]. Apart from the role of ABC transporters in cell proliferation, the disruption of ABC transporter genes also alters tumorigenesis in mouse cancer models. Disruption of the *ABCB1* gene can cause a decrease in intestinal polyps and tumour incidence [101], while the disruption of the *ABCC1* gene has been linked to reduced tumour incidence and increased tumour latency in mouse models [102]. Nevertheless, mammary tumour latency was shortened in Abcg2-deficient female *K14cre; Brca1^F/F^; p53^F/F^* mice compared with their Abcg-2 proficient counterparts [103]. Furthermore, the expression of ABC transporters might be used to predict tumour progression. *ABCC1* expression was significantly increased in grade III invasive breast ductal carcinoma tissue [104]. Similarly, *ABCC3* expression was significantly increased in grade III invasive breast ductal carcinoma tissue, as well as in chemotherapy-treated patient samples [104]. The knockdown of *ABCC1* and *ABCC3* reduced the expression of stemness genes (e.g., Nanog and Bmi1) in TNBC cell lines, and the knockdown of *ABCC3*, but not *ABCC1*, also reduced the CD44^high^/CD24^low^ breast cancer stem-like subpopulation [104]. Unfortunately, the mechanisms for ABC transporter-regulated tumorigenesis have not been established. The exact roles of ABC transporters in the transformation of normal tissues to malignant tumours have not been fully understood. A possible explanation is that the nature of ABC transporters in physiological functions may protect somatic cells from endogenous metabolites and carcinogenic xenobiotics, eventually reducing the risk of tumour evolution [105]. However, this hardly explains the pro-tumorigenic functions of ABC transporters. Referring to the value of some prognostic genes, such as *BRCA1/2* genes, in breast cancer evolution, the exploitation of ABC transporter genes in tumorigenesis is of great interest.

The expression of *ABCC11* has been linked to aggressive TNBC- and HER2-enriched breast cancer subtypes and, thus, poor prognosis in a microarray-based study involving 281 Japanese patients [106]. *ABCC11* SNP rs17822931 was strongly associated with the carcinogenic risk of ER-positive breast cancer in Japanese women [107]. This is likely due to the diminished oestrogen efflux activity with the A allele, resulting in a higher intracellular oestrogen concentration. An increased exposure to oestrogen could lead to oxidative DNA damage and, hence, carcinogenesis, especially in ER-positive tissues [107]. However, this finding is controversial, as opposite results were reported by other groups. In another study involving Japanese patients, the G allele, which causes a higher oestrogen efflux, was associated with a higher risk of breast cancer [108]. No association between rs17822931 in *ABCC11* and breast cancer risk was identified in Koreans and Europeans [109,110]. The different findings in the above studies could be due to several reasons. For example, the exposure to endogenous oestrogen might be affected by a physical condition, dietary habit and/or gene variability in different ethnicities; these factors could lead to an altered exposure to oestrogen, even in the presence of the potentially high oestrogen efflux activity caused by the *ABCC11* gene with the G allele. The involvement of other potential mechanisms needs to be identified; since the cancer cell is a complex and dynamic system, any phenotype might be a result of the collaboration between different genes. The limited sample size might also influence the breast cancer subgroup analysis. A larger number of enrolled subjects is needed in order to reduce the variance and strengthen the link between this SNP and breast cancer risk.

### 3.2. ABC Transporter and Breast Cancer Metastasis

**Figure 5:**
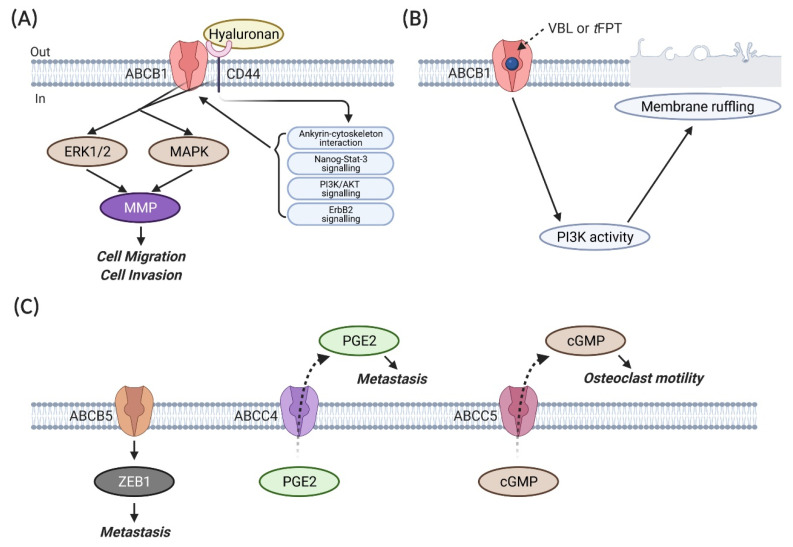
The role of ABC transporters in breast cancer metastasis. (**A**) The ABCB1–CD44 interaction leads to the activation of extracellular signal-regulated protein kinase 1/2 (ERK1/2) and p38 mitogen-activated protein kinases (MAPK), which increase metalloproteinase (MMP) at a transcriptional level and proteolytic activity and, thus, invasive behaviour [111,120]. The binding of hyaluronan to CD44 further promotes *ABCB1* expression and activity via ankyrin–cytoskeleton interactions, Nanog-Stat-3 signalling, ErbB2 signalling and the phosphatidylinositol 3-kinase/protein kinase B (PI3K/AKT)-related survival pathways [121]. (**B**) ABCB1 coupled with its substrate drug, vinblastine (VBL), or trans-flupentixol (*t*FPT), enhances the PI3K activity, which triggers membrane ruffling, an early indicator of cellular motility and metastatic potential in cancer cells [122]. (**C**) ABCB5, ABCC4 and ABCC5 regulate breast cancer metastasis via the downstream effector Zinc finger E-box-binding homeobox 1 (ZEB1) [123], the export of an inflammatory mediator prostaglandin E_2_ (PGE2) [99,124] and the efflux of cyclic guanosine monophosphate (cGMP) [125], respectively. Figures were created with BioRender.com (accessed on 22 March 2021).

Metastatic breast cancer is the major cause of cancer-related death, as aforementioned. Even though a direct linkage between ABC transporters and the metastasis of breast cancer is lacking, emerging evidence suggests the potential roles of those proteins in the cell invasion, motility and migration of breast cancer cell lines. Miletti-Gonzalez and Chen [111] found that interference with the expression or function of ABCB1 influences the motility and invasion of MCF7/AdrR breast cancer cells through interactions with CD44s (Figure 5A). CD44 is a cell surface glycoprotein of the cell adhesion molecule family [112]. The *CD44* gene encodes for more than 100 isoforms. Apart from the standard isoform CD44s, the variant isoforms of CD44, CD44v is involved in various cellular process in a cell type-dependent manner [112]. The role of CD44 in breast cancer is controversial and appears to be discrepant, relating to both poor and favourable outcomes. CD44 is known to bind to several ligands (e.g., hyaluronan, osteopontin and fibronectin) to activate genes regulating breast cancer development [113]. CD44 also interacts with other membrane proteins, such as EGFR and HER2, to promote the metastasis of breast cancer [112]. In addition, CD44 is regarded as a stemness marker of breast cancer stem cells (BCSCs) (CD44^+^/CD24^−/low^) in TNBCs [113]. It maintains the stemness and regulates the cellular adhesion, cell growth, proliferation and motility through a series of signalling pathways, such as the RhoGTPases, Ras-mitogen-activated protein kinases (MAPK) and phosphatidylinositol 3-kinase/protein kinase B (PI3K/AKT) pathways [113]. The ectopic expression of these molecular pathways may result in the transformation of normal stem cells to cancer stem cells [113]. However, CD44^+^/CD24^−/low^ BCSC abundance was found to differ in breast cancer subtypes. The CD44^+^/CD24^−/low^ phenotype was correlated to BCSCs for the more undifferentiated basal/mesenchymal cell lines, such as BT-549 and MDA-MB-231, whereas the CD44^+^/CD24^+^ phenotype was recognised as BCSCs in basal/epithelial cell lines, such as BT-20 and MDA-MB-468 [114,115]. Interestingly, another study argued that a high CD44/CD24 ratio was mainly in charge of cell proliferation and tumorigenesis in breast cancer, while the aldehyde dehydrogenase 1 (ALDH1)-positive phenotype was significantly associated with cell migration and metastasis [116]. These two markers might play different functions in breast cancer progression, indicating that the detection of a single CD44/CD24 ratio might not be enough to characterise BCSCs of breast cancer [116]. There are contradictory results in predicting BC patients’ prognosis by using the CD44^+^/CD24^−/low^ phenotype [117,118,119]. Larger clinical trials with different BC subtypes and therapeutic regimens may be needed to unravel the roles of CD44 in clinical outcomes.

The luminal breast cancer cell lines (e.g., MCF7) are reported to be enriched in the CD44^−/low^/CD24^+^ cell population [114]. Of note, *ABCB1* and *CD44s* mRNA transcripts and proteins were only detected in the resistant MCF7/AdrR cell line, rather than the sensitive parental line [111]. The expression of *ABCB1* was probably coregulated with *CD44s*, and these two proteins were colocalised. By using coimmunoprecipitation assays, ABCB1 was found to physically interact with CD44s to increase the cell migration and invasion in MCF7/AdrR cells. This increase in invasion and migration was suppressed by treating the cells with either an anti-CD44s antibody or an ABCB1 inhibitor trans-flupentixol (tFPT) at noncytotoxic concentrations or an ABCB1 substrate drug vinblastine without changes in the relative expression of ABCB1 and CD44s. These drugs could interfere with in vitro invasion and migration via promoting CD44s capping, which decreases the CD44s functionality. To test whether the expression of ABCB1 or CD44s alone influences migration, MCF7 cells (ABCB1^−^/CD44^−^) were transfected with *ABCB1* or *CD44s* cDNA-containing plasmids to obtain ABCB1 or CD44s overexpressing clones [111]. ABCB1^+^/CD44^−^ clones showed no increase in migration [111]. However, all the CD44s overexpressing clones showed the expression of the ABCB1 and MDR phenotypes against paclitaxel. The knockdown of *ABCB1* in these CD44s-overexpressing clones significantly decreased the in vitro migration [111]. These results indicate that the potential interaction between ABCB1 and CD44s affects the in vitro invasion and migration of MCF7/AdrR cells. The failure of generating ABCB^-^/CD44s^+^ transfectants and the decrease in the cell migration of *ABCB1*-silenced CD44s-overexpressing cells probably mirrors the fact that *ABCB1* expression might be coregulated with *CD44s*, and CD44s plays its role in the cell migration of breast cancer on the premise of a functional ABCB1 expression [111]. The failure of generating ABCB^−^/CD44s^+^ transfectants in the aforementioned study might be due to the hyaluronan–CD44 interaction-regulated *ABCB1* expression. It has been reported that hyaluronan–CD44 interactions are capable of stimulating *ABCB1* expression and activity through promoting ankyrin–cytoskeleton interactions, Nanog-Stat-3 signalling, ErbB2 signalling and PI3K/AKT-related survival pathways in breast cancer cell lines [121]. Thus, *ABCB1* is more like a downstream gene that is regulated by hyaluronan–CD44 interaction-activated signalling, rather than coregulated with *CD44*. Nevertheless, the role of the hyaluronan–CD44 interaction in cell invasion was contradicted in other studies; CD44 could bind to high molecular weight hyaluronan to repress the invasion of several breast cancer cell lines [126], and the loss of CD44 was significantly associated with papillary carcinoma compared with benign papilloma among 160 specimens [127]. The association between ABCB1 and CD44 was further proven by a study concluding that ABCB1 might interact with CD44 through the activation of extracellular signal-regulated protein kinase 1/2 (ERK1/2) and MAPK in the M14 ADR-resistant human melanoma cell line [120]. This activation was found to increase metalloproteinase (MMP) (MMP-2, MMP-3 and MMP-9) at a transcriptional level and proteolytic activity and, therefore, the invasive behaviour. By using fluorescence laser scanning confocal microscopy on double-immunolabeling assays, ABCB1 and CD44 were found to share the same shuttle vesicles that transport these molecules toward the plasma membrane. The knockdown of *ABCB1* by siRNA reduced the in vitro invasion and migration of M14 ADR-resistant cells [120]. Furthermore, it has been reported that, apart from the standard CD44s isoform, some other CD44 isoforms increase the invasive capability of the MCF7 breast cancer cell line through different pathways, such as CD44st through the Ras/MARK signalling pathway [128,129]. Recent studies also found that *CD44st* expression was significantly correlated with *HER2* expression and, thus, clinical outcomes in breast cancer patients. However, the mechanism remains unclear [130,131]. Besides, the observations in a study involving 124 primary breast tumour specimens showed that a high level of ABCB1 expression was associated with TNBC metastatic spread [132]. The combined genotype of *ABCC2*-24CC and *ABCB1* 3435CT or TT was related to the increased risk of bone metastases in Thai breast cancer patients treated with tamoxifen [133]. In addition, ABCB1 substrate drug vinblastine (VBL)- or trans-flupentixol (*t*FPT)-induced membrane ruffling is an early indicator of cellular motility and metastatic potential in ABCB1 overexpressing MCF7/AdrR and ABCB1 transfectant MCF7/BC-19 breast cancer cells (Figure 5B) [122]. This phenomenon was not observed in both the VBL- or *t*FPT-treated parental sensitive line (ABCB1-negative) and the non-ABCB1 substrate drug mechlorethamine-treated resistant counterpart, indicating the necessity of ABCB1 in its substrate drug-induced membrane ruffling. The observations in the immunoblotting assays suggested that this membrane ruffling induced by ABCB1 substrate drugs was related to the elevated activity of PI3K but not the protein content [122]. The protein content of PI3K remained consistent before and after treatment, but the PI3K-catalysed product phosphatidylinositol-3-phosphate (PIP) was increased 24 h after treatment [122]. The VBL- and *t*FPT-induced membrane ruffling and the PI3K activation in MCF7/AdrR cells were inhibited by the PI3K inhibitor wortmannin. However, no physical interaction between ABCB1 and PI3K was detected by the coimmunoprecipitation assays, indicating the potential involvement of other underlying mediators that need to be determined, such as the potential candidate protein kinase C [122]. These studies reveal that the exact role of ABCB1 in breast cancer cell invasion and migration is still controversial and requires more investigation. Given that ABC transporters might not be involved in hyaluronan translocation in breast cancer [134], whether the aberrant expression of *ABCB1* occurs as a result of *CD44* upregulation and whether *ABCB1* and its SNPs alter the binding activity of CD44 need to be clarified in future studies. The exact underling mechanisms also need to be identified, especially considering the opposing findings from previous studies, which could be ascribed to the *CD44* variants and *ABCB1* SNPs. The expression of CD44 is a hallmark of BCSCs (CD44^+^/CD24^−^) in some breast cancer subtypes, in which their phenotypes, such as self-renewal, metastasis, migration and therapeutic resistance, can lead to treatment failure and disease relapse. Given the potential interaction between ABCB1 and CD44, whether *ABCB1* and its SNPs are involved in the transformation of differentiated cells and normal stem cells to BCSCs is of intense interest. Further studies with larger sample sizes are warranted to determine if ABCB1 may be useful as a marker of metastatic spread.

ABCG2 expression in breast tumour cells was correlated with lymph node metastasis and clinical stages based on the analysis of invasive ductal breast carcinoma specimens from 196 patients [135]. This finding is consistent with the results derived from another study including 200 breast carcinoma specimens [136]. Nevertheless, ABCG2 expression in the tumour of TNBC patients was elevated and associated with a longer disease-free interval and OS in another study involving 124 primary breast tumour specimens [132]. Additionally, ABCG2 protein expression was significantly correlated to proliferative index Ki67 and a better OS in a cohort of TNBC patients [137]. Of note, *ABCG2* mRNA and/or protein expression were related to breast cancer side population (SP) cells in the MCF7 and MDA-MB-231 breast cancer cell lines [138]. SP cells are an aggressive subpopulation of cells that exhibit stem cell characteristics and an increased rate of Hoechst 33342 dye efflux [138]. MDA-MB-231 and MCF7 SP accounted for 2.3% and 1.9% of the total cell population and showed a 13.238- and 4.277-fold increase in ABCG2 mRNA expression, respectively. The ABCG2 protein expression remained at the same level between MDA-MB-231 SP and non-SP (NSP) cells, while MCF7 SP showed an increased ABCG2 protein expression compared with NSP. For the Fine Needle Aspirate (FNA) samples collected from 49 palpable breast tumours, SP cells were detected in 12 samples at various percentages, from 0.4% to 3.9% [138]. Within the 12 tumour samples with the presence of SP cells, SP cells were more frequently detected in ER-negative and TNBC breast tumours. ABCG2 protein expression also displayed a significant increase in TNBC samples compared with other subtypes, but not all patient-derived SP cells exhibited ABCG2 transcripts. These results indicate that ABCG2 expression might be prevalent in SP cells but may not be a suitable biomarker for screening SP cells in breast cancer [138]. However, SP cells were related to ER-positive breast cancer in another study conducted by Nakanishi and Chumsri [139]. The authors found that ABCG2 membrane positivity showed a two- to three-fold increase in SP cells in both MCF7/HER2-18 *HER2* cDNA-transfected cells (ER-positive) and patient-derived GCC-BC4 cells (ER-positive) compared with NSP cells [139]. However, these results might be influenced by the specimens used for the analysis. The random integration of *HER2* cDNA-containing plasmids into the chromosome of MCF7 cells may lead to the aberrant expression of other genes. There is a risk that the signalling pathways involved in ER expression are compromised by this phenomenon. Additionally, the SP characteristics of the SP cells isolated from primary human breast cancer in this study might be changed after several rounds of subculture. Despite the discrepancy of ABCG2 with SP cells in breast cancer, determining if ABCG2 or other ABC transporters are responsible for the SP phenotype is of great importance. This may help predict the tumour response to chemotherapeutics and the risk of relapse in breast cancer patients.

Apart from ABCB1 and ABCG2, some other ABC transporters are also implicated in breast cancer metastasis. Yao and Yao [123] found that ABCB5 may enhance the metastasis and epithelial–mesenchymal transition (EMT) in breast cancer through the downstream effector Zinc finger E-box-binding homeobox 1 (ZEB1) (Figure 5C). The knockdown of ABCB5 or ZEB1 reversed the ABCB5 ectopic expression-induced cell invasion, migration and EMT [123]. ABCC1 could promote the migration of MCF7 cells through the export of S1P (Figure 4A). It was also related to enhanced murine breast tumour tumorigenesis and growth and a shorter survival of mice bearing tumours overexpressing ABCC1 [100]. A higher staining intensity of ABCC1 was observed in lymph node metastasis than in the primary tumours of breast cancer patients [140]. The knockdown of *ABCC4* led to a decreased metastatic potential in TNBC cells, suggesting its role in breast cancer metastasis [99,124]. This might be due to the efflux of the inflammatory mediator prostaglandin E_2_ (PGE2) mediated by ABCC4 (Figure 5C). The increased level of PGE2 in the tumour microenvironment was associated with the activation of several signalling pathways relating to breast cancer metastasis [124]. Both the mRNA and protein levels of *ABCC5* were found to be related to osteoclast formation and breast cancer skeletal metastases (Figure 5C) [125]. The substrate of ABCC5 may either promote osteoclastogenesis directly or stimulate this process indirectly through an intermediate cell type present in the bone microenvironment. It was postulated that the locally elevated level of cGMP that is pumped out by ABCC5 can further increase the osteoclast motility [125]. Taken together, a large cohort of ABC transporters are potentially involved in breast cancer metastasis. However, the demonstrated abilities of ABC transporters appear to be discrepant. These controversial observations might be derived from the different gene variants and breast cancer subsets used in the respective studies. Additionally, as the metastases of cancer cells are believed to depend on disseminated tumour cells [141] and circulating tumour cells [142] that shed into the blood circulation, whether the expression of ABC transporters are conserved in the primary tumour, circulating tumour cells and the distant metastases needs to be comprehensively investigated. Breast tumour behaviour is thought to be driven by many different factors, while the heterogeneity of breast tumours makes it more like a “multi-disease”. Thus, the role of ABC transporters and their isoforms in BCSCs, SP cells and differentiated cells might need to be carefully classified and investigated. Given that the recognition of the signalling pathways and molecular mechanisms involved in cancer progression could be effective for cancer treatment, the cell surface localisation and the potential roles of ABC transporters in cancer chemoresistance, progression and metastasis make these membrane proteins a promising target for further investigation.

## 4. Conclusions

Breast cancer is the most commonly diagnosed cancer and the leading cause of death in female patients. There is no doubt that the early detection of breast cancer is always a good practice, as it results in better outcomes. The breakthrough in the development of noninvasive body fluid-based tests (e.g., blood, urine, tears, sweat and breath) supplemented with the current clinical approaches has a striking potential to increase the precision and accuracy of the earlier detection of breast cancer [143]. Nevertheless, in patients who fail to be diagnosed early and develop MDR, clinical MDR seems to be independent of the ABC transporter expression, even though various in vitro examples displayed that ABC transporters probably affect the tissue distribution of many chemotherapeutics and directly pump them out to render a chemoresistance. Accumulating evidence also defines the role of some ABC transporters in breast cancer cell proliferation, invasion and migration, but this is contradicted by other studies. These controversial findings might be caused by the limitations in the detection method, gene fusion status and SNPs and failure in classifying the molecular characteristics and subsets of breast cancer. Previous attempts were made to develop ABC transporter inhibitors for clinical use to overcome MDR, but they failed in clinical trials primarily because the trials did not limit the patient selection to those whose tumours were nonresponsive to treatment due to ABC transporter overexpression. Although the clinical inhibition of ABC transporters is disappointing and their prognostic value in breast cancer is conflicting, by overcoming the above obstacles, the modulation of ABC transporters may eventually represent a promising strategy to prevent the chemoresistance, progression and metastasis of breast cancers. Novel preclinical models (e.g., breast tumour organoids), together with a targeted drug delivery system, are expected to identify novel approaches to antagonise/modulate ABC transporters. Future clinical perspectives may include screening the tumour expression of ABC transporters to select favoured patients for treatments with standard regimens or in combination with a potent and specific inhibitor.

## Figures and Tables

**Table 1:** Anticancer drug substrates of the ATP-Binding Cassette (ABC) transporters.

Names	Substrates	References
ABCB1	Olaparib, Doxorubicin, Epirubicin, Docetaxel, Paclitaxel, Cisplatin, 5-Fluorouracil, Palbociclib, Ribociclib, Abemaciclib, ICEC0942, THZ1, Larotrectinib, Tivozanib, Galunisertib, Fisogatinib, Osimertinib, Actinomycin D, Bisantrene, Dasatinib, Daunorubicin, Digoxin, Etoposide/VP-16, Homoharringtonine, Irinotecan, Mitoxantrone, Teniposide, Topotecan, Vinblastine, Vincristine, Vindesine, Vinorelbine, Methotrexate, Mithramycin Mitomycin C	[14,27,29,30,31,32,33,58,65,69]
ABCC1	Doxorubicin, Epirubicin, Daunorubicin, Eribulin, Chlorambucil, Idarubicin, Etoposide/VP-16, Glucuronide, Teniposide, Vincristine, Vinblastine, Vinorelbine, Topotecan, Irinotecan/SN-38, Mitoxantrone, saquinavir, Arsenite, Trivalent antimony, Imatinib, Melphalan, Methotrexate, SN-38	[14,27]
ABCC2	Doxorubicin, Epirubicin, Paclitaxel, Carboplatin, Cisplatin, Etoposide/VP-16, Irinotecan, Irinotecan/CPT-11, Methotrexate, Mitoxantrone, saquinavir, SN-38, Sulfinpyrazone, Topotecan, Vinblastine, Vincristine	[14,72]
ABCC3	Methotrexate, Etoposide/VP-16, Teniposide, Vincristine	[14,73]
ABCC4	Methotrexate, 5-Fluorouracil, 6-mercaptopurine, 6-thioguanine, Bisantrene, Irinotecan/CPT11, Nucleoside monophosphates, Topotecan, Vinblastine, SN-38	[14,73,74]
ABCC5	Doxorubicin, Gemcitabine, Methotrexate, 5-Fluorouacil, 6-mercaptopurine, 6-thioguanine, Bisantrene, Mitoxantrone, Nucleoside monophosphates	[14,73]
ABCG2	Doxorubicin, Epirubicin, Methotrexate, Palbociclib, Abemeciclib, THZ1, Larotrectinib, Tivozanib, Osimertinib, Bisantrene, Dasatinib, Docetaxel, Daunorubicin, Flavopiridol, Irinotecan/CPT-11, Mitoxantrone, Nilotinib, SN-38, topotecan, Tyrosine Kinase inhibitors	[14,29,30,32,69,75,76,77]

## Data Availability

Publicly available datasets were analyzed in this study. This data can be found here: http://www.proteinatlas.org; Last access date: 1 March 2021.

## Abbreviations

ABC	ATP-binding cassette
ABCB1	ABC subfamily B member 1
ADP	Adenosine diphosphate
AKT	Protein kinase B
ALDH1	Aldehyde dehydrogenase 1
ATP	Adenosine Triphosphate
BBB	Blood-brain barrier
BCRP	Breast cancer resistance protein
BCSC	Breast cancer stem cell
BPD	Benzoporphyrin derivative
cAMP	Cyclic adenosine monophosphate
cGMP	Cyclic guanosine monophosphate
CD44-ICD	Intracellular domain fragment of CD44
CS	Collateral sensitivity
CSF	Blood-cerebrospinal-fluid barrier
DFS	Disease-free survival
EMT	Epithelial-to-mesenchymal transition
ERK1/2	Extracellular signal-regulated protein kinase 1/2
FAC	5-fluorouracil, Adriamycin and cyclophosphamide
FEC	5-fluorouracil, epirubicin and cyclophosphamide
HGSC	High-grade serous ovarian cancer
HR	Homologous recombination
MAPK	Mitogen-activated protein kinases
MBC	Metastatic breast cancer
MDR	Multidrug resistance
MDR1	Multidrug resistance protein 1
MMP	Metalloproteinase
MRP1	MDR-associated protein 1
NBDs	Nucleotide-binding domains
NSP	Non-side population
NTRK	Neurotrophic tropomyosin receptor kinase
OS	Overall survival
P-gp	P-glycoprotein
PARP	Poly (ADP-ribose) polymerase
PDT	Photodynamic therapy
PFS	Progression-free survival
PI3K	Phosphatidylinositol-3-kinase
PIP	Phosphatidylinositol-3-phosphate
PKA	protein kinase A
S1P	Sphingosine-1-phosphate
SP	Side population
SNPs	Single nucleotide polymorphisms
SPAG5	Sperm-associated antigen 5
TAM	Tamoxifen
TMDs	Transmembrane domains
ZEB1	Zinc finger E-box-binding homeobox 1
